# Unsupervised Adaptive Weight Pruning for Energy-Efficient Neuromorphic Systems

**DOI:** 10.3389/fnins.2020.598876

**Published:** 2020-11-12

**Authors:** Wenzhe Guo, Mohammed E. Fouda, Hasan Erdem Yantir, Ahmed M. Eltawil, Khaled Nabil Salama

**Affiliations:** ^1^Sensors Lab, Advanced Membranes & Porous Materials Center, Computer, Electrical and Mathematical Sciences and Engineering Division, King Abdullah University of Science and Technology, Thuwal, Saudi Arabia; ^2^Communication and Computing Systems Lab, Computer, Electrical and Mathematical Sciences and Engineering Division, King Abdullah University of Science and Technology, Thuwal, Saudi Arabia; ^3^Department of Electrical Engineering and Computer Science, University of California, Irvine, Irvine, CA, United States

**Keywords:** neuromorphic computing, spiking neural networks, pruning, unsupervised learning, STDP, pattern recognition

## Abstract

To tackle real-world challenges, deep and complex neural networks are generally used with a massive number of parameters, which require large memory size, extensive computational operations, and high energy consumption in neuromorphic hardware systems. In this work, we propose an unsupervised online adaptive weight pruning method that dynamically removes non-critical weights from a spiking neural network (SNN) to reduce network complexity and improve energy efficiency. The adaptive pruning method explores neural dynamics and firing activity of SNNs and adapts the pruning threshold over time and neurons during training. The proposed adaptation scheme allows the network to effectively identify critical weights associated with each neuron by changing the pruning threshold dynamically over time and neurons. It balances the connection strength of neurons with the previous layer with adaptive thresholds and prevents weak neurons from failure after pruning. We also evaluated improvement in the energy efficiency of SNNs with our method by computing synaptic operations (SOPs). Simulation results and detailed analyses have revealed that applying adaptation in the pruning threshold can significantly improve network performance and reduce the number of SOPs. The pruned SNN with 800 excitatory neurons can achieve a 30% reduction in SOPs during training and a 55% reduction during inference, with only 0.44% accuracy loss on MNIST dataset. Compared with a previously reported online soft pruning method, the proposed adaptive pruning method shows 3.33% higher classification accuracy and 67% more reduction in SOPs. The effectiveness of our method was confirmed on different datasets and for different network sizes. Our evaluation showed that the implementation overhead of the adaptive method regarding speed, area, and energy is negligible in the network. Therefore, this work offers a promising solution for effective network compression and building highly energy-efficient neuromorphic systems in real-time applications.

## Introduction

In recent years, as the prediction of Moore’s law slows down prominently, neuromorphic computing has been widely regarded as a promising approach for large-scale computing. Neuromorphic systems are constructed following biological principles existing in our central nervous systems, which features in massive parallelism, collocated memory, and processors, and asynchronous event-driven computation (Mead, 1990; Furber et al., 2014; Davies et al., 2018).

Generally considered as the third generation of neural network models, spiking neural networks (SNNs) have started a paradigm shift in the brain-inspired research exploration. Different from artificial neural networks (ANNs), SNNs are well-known for its capability of accurately capturing neural dynamics and biological behaviors of the central nervous system and processing spatio-temporal information. With energy-efficient computation and parallel information processing features, SNNs are widely adopted for building neuromorphic hardware systems (Thakur et al., 2018). In such systems, information is transmitted through synapses from a presynaptic neuron to a postsynaptic neuron on the occurrence of an event (or a spike). Neural networks require deep and complex structures to tackle real-world tasks, like pattern recognition, object detection, and motor controls (Shrestha and Mahmood, 2019). The complexity leads to large synaptic memories and high energy consumption, which poses a big challenge in hardware implementation. Therefore, it is necessary to search for practical solutions to reduce network complexity and improve the energy efficiency of SNNs.

During early brain development, creations of synaptic connections between neurons exponentially increase with the numerous stimuli coming from environments every day (Zillmer and Spiers, 2001). The rapid synapse creation is vital for learning and memory formation. Between early childhood and adulthood, weight pruning occurs as a natural process during which our brain eliminates unnecessary synaptic connections. It is regarded as a purposeful process of maintaining a more efficient brain function. This biological process has been extensively studied in current ANNs for its attractive memory and energy reduction benefits. Han et al. (2015) introduced a training-pruning-retraining approach that can reduce the number of synaptic connections by 12x and computational operations by 5x for the VGG-16 network. Weight pruning was also proved to be an effective means of alleviating the overfitting problem in ANNs (Paupamah et al., 2020). Moreover, to avoid irregular structure of pruned weight matrices and aid in the leverage of sparse matric-vector multiplication, a variety of structured weight pruning techniques were proposed where the entire rows and columns in the weight matrices are removed by imposing certain constraints during the pruning process (Anwar et al., 2017; Sredojevic et al., 2017).

While weight pruning has been widely applied in different ANNs, the benefits that weight pruning could provide for SNNs have yet to be explored. Limited works have reported applying weight pruning in SNNs so far (Iglesias et al., 2005; Rathi et al., 2019; Shi et al., 2019). Rathi et al. proposed a spike-timing-dependent plasticity (STDP) based online synaptic pruning method, which sets non-critical weights to zero during the training phase and removes the weights below a certain threshold at the end of training (Rathi et al., 2019). This method only sets the weights to zero without removing them. It allows them to be updated during training, which is not an effective approach to improve the energy efficiency for online learning systems. Shi et al. presented an online soft-pruning method by setting the weights below a constant threshold to a constant value instead of removing them during training. While this method could reduce the number of STDP updates during training, it does not induce any sparsity in the network, leading to little benefit for hardware implementation. Moreover, these pruning methods use a constant weight threshold throughout the whole pruning process. With a constant threshold, the network can not effectively select the non-critical weights to be pruned. In the early phase of training, weights are not completely learned, and a large threshold can mistakenly remove important weights. If a small threshold is used, some non-critical weights can not be pruned at the end of training since these weights could grow. On the other hand, the connection strength of neurons in one layer with the previous layer varies. A large threshold could remove most of the critical weights from the neurons with weak connection and hence severely affect the neurons’ function, which could lead to substantial performance degradation of the network. Therefore, it is crucial to adapt the weight threshold over time and all the neurons during training to improve network performance. In this work, we propose an online adaptive weight pruning method that adapts the pruning threshold over time and neurons during training and completely remove the weights below the threshold from the network. It is demonstrated to be an effective approach for reducing network complexity and improving energy efficiency during both training and inference operations.

The main contributions of this work are summarized as follows.

• A simple online adaptation scheme for the pruning threshold is presented, which can change the threshold dynamically over time during training. It also considers the spatial difference of the connection strength of neurons in one layer with the previous layer and adapts the threshold over the neurons based on their firing activity.
• The proposed method is demonstrated to be more effective in retaining classification accuracy after pruning than the constant threshold weight pruning and neuron pruning methods. It resulted in a 67% reduction in synaptic operations (SOPs) while outperforming the previously reported soft weight pruning method by 3.33%. The advantage of the proposed method was confirmed in the SNN on different datasets with different network sizes.
• In terms of training, the proposed online adaptive pruning method outperformed post-training pruning methods by providing more than 30% reduction in training SOPs when the pruning percentage is larger than 90% while providing more than 3% higher classification accuracy, which shows significant potential for developing high-performance and energy-efficient online neuromorphic learning system.
• The overhead of implementing the proposed method in a neuromorphic system is demonstrated to be insignificant in terms of processing speed, area, and energy.

This paper is organized as follows: section “Methods and Results” introduces different neural models used in this work and the SNN architecture. It then presents an overview of our methods, algorithmic implementation details, and pruning results for each method. In section “Comparisons and Discussions,” different pruning methods are discussed and compared. Section “Conclusion” concludes this work.

## Methods and Results

### Network Models and Architecture

To model spiking neurons, the leaky integrated-and-fire (LIF) model was used in this work because of its computational efficiency and capability of capturing the essential features of information processing in the nervous system (Burkitt, 2006). The model consists of one first-order linear differential equation that defines the dynamics of membrane potential where synapses are modeled as conductance, as described by

(1) $$\tau_{m}\,\frac{d\,v}{d\,t}=\left(v_{r}-v\right)-g_{e}\,\left(v-E_{e\,x\,c}\right)-g_{i}\,\left(v-E_{i\,n\,h}\right)$$

where τ_*m*_ is the time constant, *v*_*r*_ is the resting membrane potential, *g*_*e*_ is the excitatory conductance associated with an excitatory channel, *E*_*exc*_ is the reverse potential of the channel, *g*_*i*_ is the conductance associated with an inhibitory channel, *E*_*inh*_ is the reverse potential of the channel. The model resets the membrane potential to *v*_*r*_ and generates a spike if the membrane potential reaches a defined threshold *v*_*th*_. Synaptic conductance follows a time-varying dynamics governed by Diehl and Cook (2015),

(2) $$\tau_{g}\,\frac{d\,g}{d\,t}=-g+\sum_{j}w_{i\,j}\,\delta \,\left(t-t_{j}^{f}\right)$$

where *g* is the conductance, τ_*g*_ is the time constant, *w*_*ij*_ is the synaptic weight from the presynaptic neuron *j* to the postsynaptic neuron *i*, and $t_{j}^{f}$ is the firing time of the presynaptic neuron *j*.

Spike-timing-dependent plasticity relates the synaptic plasticity to the relative timing difference between a presynaptic spike and a postsynaptic spike. A triplet-based STDP model was used in this work because of its biological plausibility and easy implementation (Pfister and Gerstner, 2006). It overcomes the limitation of the paired-based STDP models to accommodate the dependence on the repetition frequency of the pairs of spikes. It was shown that the triplet rule is more biological plausible where its response can fit the experimental data from the visual cortical slices and hippocampal cultures (Pfister and Gerstner, 2006). The model considers sets of three spikes (one presynaptic and two postsynaptic spikes), each of which leaves a time-varying trace whose dynamics are described below.

(3) $$\frac{d\,s}{d\,t}=-\frac{s}{\tau_{s}},s\in \left\{x_{j},y_{i}^{1},y_{i}^{2}\right\}$$

where *x*_*j*_ is the trace variable associated with the firing event of the presynaptic neuron *j*, $y_{i}^{1}$, and $y_{i}^{2}$ are the fast and slow trace variables associated with the firing event of the postsynaptic neuron *i*, respectively, and τ_*s*_ is the corresponding time constant. When the presynaptic neuron (or postsynaptic) fires, the related trace *x*_*j*_ (or *y*_*i*_) is reset to 1. The weight updates are carried out as below.

(4) $$\Delta \,w_{i\,j}=\left\{ \begin{array}{cc} -\mu_{p\,r\,e}\,y_{i}^{1}, & \mathrm{if}\,\mathrm{the}\,\mathrm{neuron}\,i\,\text{fires},\\ +\mu_{p\,o\,s\,t}\,x_{j}\,y_{i}^{2}, & \mathrm{if}\,\mathrm{the}\,\mathrm{neuron}\,j\,\text{fires}. \end{array}\right.$$

where μ_*pre*_ and μ_*post*_ are the corresponding weight updating rates.

**FIGURE 1:**
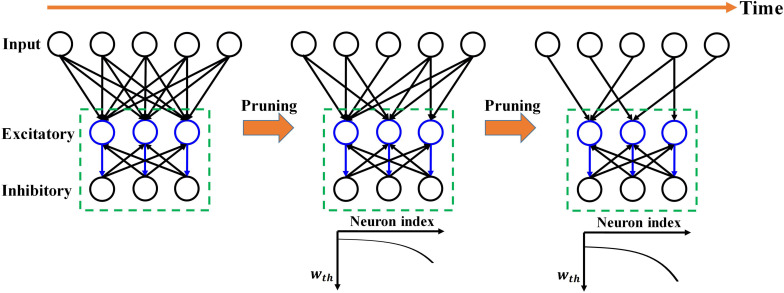
Overview of the proposed adaptive pruning process in SNNs. The SNN architecture consists of two layers, an input layer and a winner-take-all (WTA) layer with excitatory and inhibitory neurons connected to each other. Pruning only happens in the synapses from the input layer to the WTA layer. *w*_*th*_ is the weight pruning threshold.

In this work, a two-layer SNN architecture was adopted, as shown in Figure 1, and tested on the Modified National Institute of Standards and Technology (MNIST) dataset and Fashion-MNIST dataset (Lecun et al., 1998; Xiao et al., 2017). This architecture consists of an input layer and a processing layer. The input layer has 784 units, each of which receives the corresponding pixel in a digit image from the MNIST dataset and produces a Poisson spike train with a frequency proportional to the pixel intensity. This encoding scheme is commonly referred to as rate coding (O’Connor et al., 2013). The input layer is fully connected to the processing layer. In the processing layer, excitatory neurons send spikes to inhibitory neurons in a one-to-one fashion, whereas each inhibitory neuron sends spikes to all the excitatory neurons except the one that it receives spikes from. This connection pattern implements a winner-take-all (WTA) mechanism, which imposes lateral inhibition on excitatory neurons and hence competitions for learning input features. To ensure fair competition, a threshold adaptation scheme is applied. Whenever a neuron fires, its threshold is increased by an adaptation constant and then slowly decays with time. The phenomenon of threshold adaptation has been commonly observed in the central nervous system (Fontaine et al., 2014). In this work, the networks with 100 and 800 excitatory neurons were used to verify the effectiveness of our proposed pruning method. A simple classification scheme is implemented based on the firing activity of excitatory neurons. After training, excitatory neurons are assigned labels to which they fire the most spikes. They are then divided into ten groups, each of which corresponds to a digit and contains all the neurons labeled by this digit. During inference, the classification result for an input image is the digit of the group with the highest average spike counts. The model parameters used in the simulation are listed in Table 1. The parameters were configured through a genetic algorithm to achieve the best accuracy. The classification accuracy on MNIST dataset achieved in these two SNNs without pruning is 85.78%/90.40%, respectively, while the accuracy on Fashion-MNIST dataset is 64.57%/69.21%, respectively. All the simulations in this work were run in a Python-based platform.

**TABLE 1:** Model parameters used in the simulation.

Model parameters	Description	Value
τ_*m*_,τ_*ge*_,τ_*gi*_	Time constants in the LIF model	100 ms, 1 ms, 2 ms
*v*_*r*_,*v*_*th*_,*E*_*exc*_,*E*_*inh*_	Potential constants in the LIF model	−60 mV, −50 mV, 0, −100 mV
τ_*x*_,τ_*y*1_,τ_*y*2_	Time constants in the STDP model	8 ms, 16 ms, 32 ms
μ_*pre*_,μ_*post*_	Learning rates in the STDP model	0.0001, 0.01
θ	Threshold adaptation constant	0.01 mV

**TABLE 2:** Comparison among different pruning methods in the SNN trained on MNIST dataset.

Pruning methods	Accuracy loss 100/800	Training SOPs reduction 100/800	Inference SOPs reduction 100/800
Online adaptive neuron pruning Guo et al., 2020	38.64%/38.85%	33%/48%	90%/90%
Post-training weight pruning Rathi et al., 2019	9.43%/9.21%	0%/0%	70%/71%
Online soft weight pruning Shi et al., 2019	12.45%/5.94%	25%/24%	0%/0%
Online constant weight pruning Shi et al., 2019	13.23%/6.73%	46%/46%	70%/69%
Online adaptive weight pruning (Our work)	6.73%/3.87%	30%/36%	69%/68%

*Accuracy loss and SOPs reduction for two network sizes (100 and 800 neurons) are shown. The connectivity is selected as 10%.*

### Overview of the Proposed Pruning Methods

Pruning is a natural process existing in human brains to maintain their efficient function. It is widely adopted in neural networks to reduce network complexity and improve energy efficiency. Various works have demonstrated the practical effectiveness of weight pruning in reducing the number of parameters and computational operations without losing accuracy (Han et al., 2015; Li et al., 2019; Tung and Mori, 2020). For example, Li et al. compressed different deep neural networks using weight pruning on mobile devices for real-time applications. They showed significant memory storage reduction and speedup. Pruning is commonly applied after training, which is suitable for improving the energy efficiency of inference systems with offline training. Pruning while training technique has been proved to be very useful in SNNs to improve online learning systems that can learn and infer the real-world information (Rathi et al., 2019; Shi et al., 2019).

However, in the previously reported online pruning methods, pruning was conducted with a constant threshold for all the synaptic weights during training. It is not an effective approach to select the non-critical weights since weights change over time during training. A large threshold can mistakenly remove many important weights at the beginning of training and severely affect the functions of the neurons with weak synaptic connections, leading to substantial performance degradation of the network, while a small threshold is not able to remove some non-critical weights at the end. In this work, we will present different pruning while training methods by adapting the pruning threshold over time and neurons. The overview of the proposed pruning process is depicted in Figure 1, where the pruning process progresses during training. Pruning is carried out only in the synapses between the input and excitatory layers since only these synaptic weights are plastic and subject to training. Initially, the network has a fully-connected structure between the input layer and the WTA layer. When the pruning process starts, the pruning threshold (*w*_*th*_) is adapted and remains different for all the excitatory neurons according to their firing activity. Fewer weights are removed for the neurons with lower thresholds. Moreover, over time, the threshold for each neuron is increased so that more weights are pruned at later pruning stages.

**FIGURE 2:**
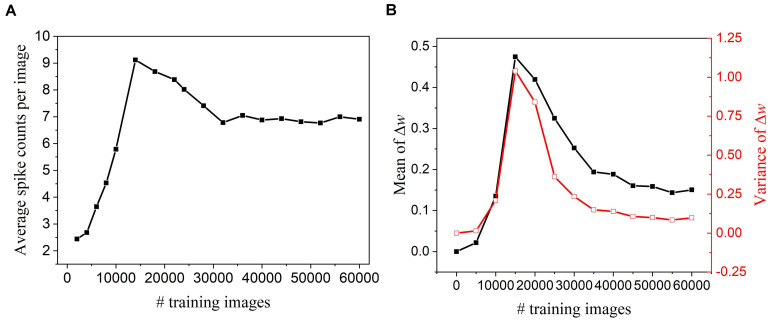
Network dynamics were monitored every 5,000 training images in the SNN with 100 excitatory neurons during training and without pruning. **(A)** Firing activity. The average of spike counts of 100 excitatory neurons was calculated. **(B)** Statistics of weight updates Δ*w* with the mean (black) and variance (red).

To perform pruning while training effectively, we need to determine when to start the pruning process. If the pruning process starts too early, important weights that have a profound impact on the output could be mistakenly pruned away, which will deteriorate network performance. On the other hand, if it starts too late, the network might not have enough training cycles to compensate for the accuracy loss and reduced improvement in training energy efficiency. To find this critical point, we have observed how the network dynamics evolve with time by monitoring firing activities and weight updates of excitatory neurons. Figure 2A shows that neurons start to fire regularly after training over 30,000 images, suggesting that the network has learned the major input features and starts to adjust for small details. In Figure 2B, the statistics (mean and variance) of weight updates over time have also revealed the same network behavior. As a result, the pruning process was decided to start after training over 30,000 images. Moreover, the pruning process was performed in multiple steps by dividing the whole dataset into multiple batches. The batch size was selected as 5,000 under the consideration of pruning frequency. The detailed implementation and algorithm of the proposed pruning methods are presented and discussed in the following sections.

### APT: Online Adaptive Pruning Over Time

**FIGURE 3:**
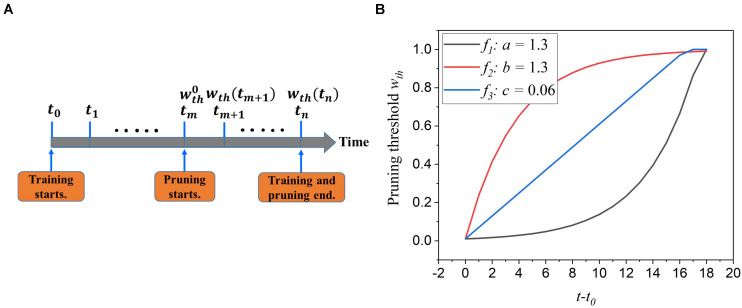
**(A)** The illustration of the online adaptive pruning scheme over time. $w_{t\,h}^{0}$ is the initial pruning threshold and *w*_*th*_(*t*) is the pruning threshold at time *t*. **(B)** The evolution of the pruning threshold over time with $w_{t\,h}^{0}$ set as 0.036.

During training, synaptic weights are randomly initialized and updated according to the input features over time. Some weights approach high value and contribute largely to network performance, whereas some are reduced to zero and less critical. The first adaptation scheme is to adapt the pruning threshold over time during training. The pruning process is illustrated in Figure 3A. The pruning starts after training over 30,000 images at a time *t*_*m*_ and an initial pruning threshold $w_{t\,h}^{0}$ is given. It ends when the training process is finished. This scheme is to increase the pruning threshold with time. The motivation behind it is to allow more weights to be trained and avoid removing critical weights mistakenly at the early training phase. At the end of the training, weights are already trained enough, and a larger threshold will not significantly increase the chance of critical weights being pruned unintentionally. The threshold adaptation scheme can be formularized by *w*_*th*_(*t*) = *f*(*t*), where *w*_*th*_(*t*) is the purning threshold at time *t*, and *f*(*t*) is the adaptation function. To select a suitable adaptation function, we propose two different exponential functions (*f*_*1*_ and *f*_*2*_) and a linear function (*f*_*3*_), described below.

(5) $$f_{1}\,\left(t\right)=w_{t\,h}^{0}\,a^{t-t_{m}},$$

(6) $$f_{2}\,\left(t\right)=w_{m\,a\,x}-\left(w_{m\,a\,x}-w_{t\,h}^{0}\right)\,b^{-\left(t-t_{m}\right)},$$

and

(7) $$f_{3}\,\left(t\right)=w_{t\,h}^{0}+c\,\left(t-t_{m}\right)$$

where $w_{t\,h}^{0}$ is the initial pruning threshold at the starting pruning time *t*_*m*_, *w*_*max*_ is the maximum value of weights, *a*,*b*, and *c* are the corresponding adaptation factors in the functions. These three functions are all confined in the range $\left[w_{t\,h}^{0},w_{m\,a\,x}\right]$. It is worth noting that the pruning threshold has to be less than *w*_*max*_ to avoid pruning the entire network. Figure 3B shows how these functions change the pruning threshold over time. Clearly, the *f*_*1*_ function increases the threshold slowly at the beginning and rapidly at the end, while the *f*_*2*_ function has the opposite effect. The linear function (*f*_*3*_) keeps the same updating rate.

**FIGURE 4:**
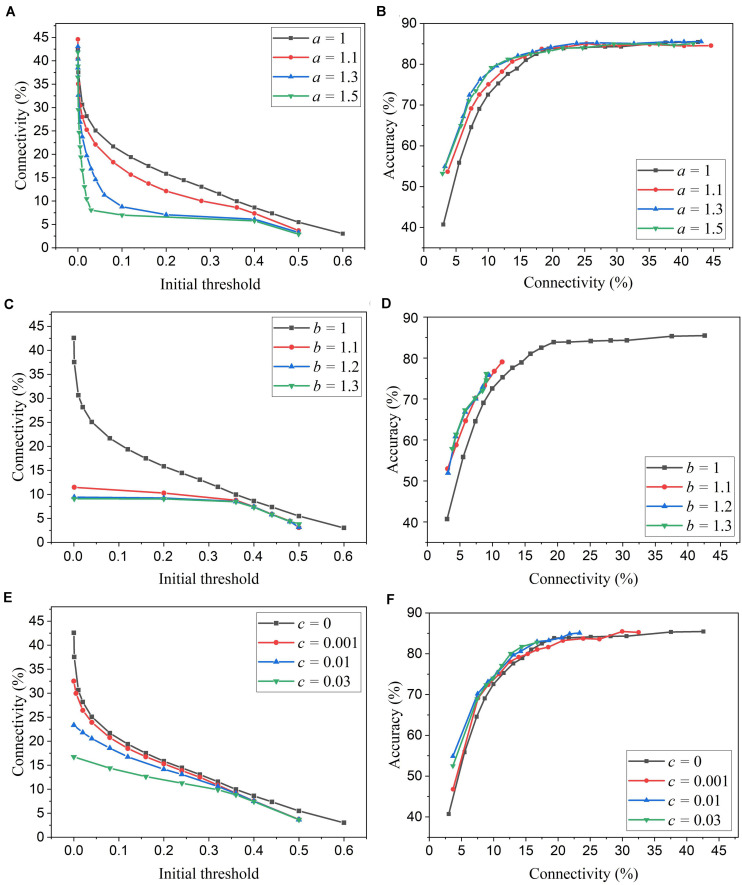
Simulation results of the online adaptive pruning over time for different adaptation functions in the SNN trained on MNIST dataset with 100 excitatory neurons. **(A,C,E)** show the network connectivity changes with the initial threshold for the adaptation functions *f*_*1*_, *f*_*2*_, and *f*_*3*_, respectively. **(B,D,F)** present the accuracy changes with the network connectivity for the adaptation functions *f*_*1*_, *f*_*2*_, and *f*_*3*_, respectively. The connectivity is defined as the percentage of the unpruned weights in the total weights.

We simulated SNNs with the proposed online pruning method. Online pruning with a constant threshold was also included as a reference to demonstrate the effectiveness of our proposed pruning methods and will be referred to as online constant pruning hereinafter. Figure 4 shows the simulation results of SNNs with the online adaptive pruning over time for three different adaptation functions, namely *f*_*1*_, *f*_*2*_, and *f*_*3*_, as described above. For each adaptation function, the results of network connectivity vs. initial threshold (*w*_*th*_(*t*_0_)) and accuracy vs. connectivity are presented, where the connectivity is defined as the percentage of non-zero weights in the total weights. The impact of different values of the corresponding adaptation factors (*a*, *b*, and *c*) is also studied. In Figures 4A,C,E, it can be seen that different initial thresholds result in different connectivity levels, and the higher the threshold, the smaller the connectivity. It should be noted that for *a* = 1, *b* = 1, and *c* = 0, they all are equivalent to the constant pruning case. By increasing the corresponding adaptation factors, a smaller initial threshold is needed to reach certain connectivity. In Figure 4C, with the factor *b* > 1, the connectivity becomes very small (<15%) even when a very low threshold is used. This is because the adaptation function *f*_*2*_ increases the threshold value very rapidly at the early phase of pruning process and hence results in a high threshold most of the time, as shown in Figure 3B. In Figures 4B,D,F, the accuracy decreases with the connectivity, as synaptic weights are pruned, and the network becomes sparse. We use the accuracy vs. connectivity as a performance metric to compare these adaptation functions and different pruning methods since pruning aims to reduce network complexity and maintain high classification accuracy. A network with high accuracy and small connectivity is desired. By applying adaptation over time (the adaptation factor >1), performance improvement can be observed for all the three adaptation functions. In Figure 4B, for the function *f*_*1*_, *a* = 1.3 is slightly better than *a* = 1.5 and hence selected as the optimized value for *a*. In Figure 4D, for the function *f*_*2*_, all the three cases (>1) have similar overall performance, but *b* = 1.1 is able to reach higher accuracy with larger connectivity and thus selected as the optimized value for *b*. In Figure 4F, for the function *f*_*3*_, *c* = 0.01 shows the best overall performance and thus is selected.

### APN: Online Adaptive Pruning Over Neurons

The excitatory neurons in the network play different roles in contributing to network performance. The connection strength of these neurons to the input layer is different. A stronger connection makes the neuron more resilient to pruning, whereas a weaker connection makes the neuron more susceptible. Applying a constant threshold for all the neurons can not effectively take the difference into consideration, and a large threshold can significantly deteriorate the function of the neurons with a weak connection. Thus, we propose an adaptation scheme over neurons by adapting the pruning threshold over all the excitatory neurons. The aim is to ensure that a smaller threshold is applied for weaker neurons and a larger threshold is for stronger neurons. So, we can balance the connection strength of all the neurons after pruning to achieve large network sparsity and maintain high classification accuracy.

**FIGURE 5:**
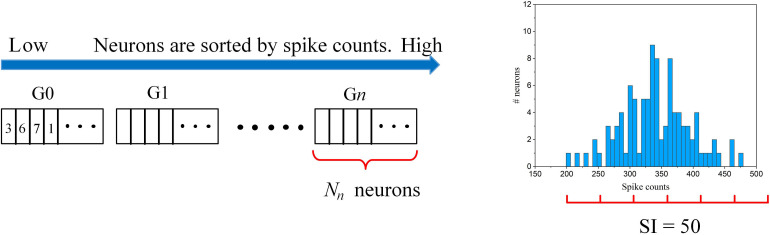
The illustration of the online adaptive pruning scheme over neurons. $w_{t\,h}^{n}$ is the pruning threshold for the *n*-th group (G*n*). *N*_*n*_ is the number of neurons in the *n*-th group. SI = 50 is used as an example for demonstrating the grouping method based on an example spike count distribution.

The adaptation scheme is illustrated in Figure 5. The connection strength of each neuron to the input layer can be reflected by the firing activity. The more the neuron fires, the stronger connection it has to the input. So, the neurons are ranked according to their spike counts and divided into multiple groups. The spike count of each neuron was calculated as the average spike count during one batch training. Each group shares the same pruning threshold, and the threshold increases along from the first group (G0) to the *n*-th group (G*n*). The grouping scheme is explained as follows. Firstly, a spike count interval is defined as *SI*. Starting from the neuron with the minimum spike count, we group all the neurons with spike counts within [*S*,*S* + *SI*], where *S* is the minimum spike count of the ungrouped neurons. In this way, the neurons in the same group have a spike count difference not larger than *SI*, so we can fairly sort the neurons with similar connection strength into one group. Across all the groups, the pruning threshold $w_{t\,h}^{n}$ is adapted according to an adaptation function *f*(*n*), where *n* is the group index. In Figure 5, an example of the grouping process is shown where an example spike count distribution and SI = 50 are used. The neurons with spike counts that fall into an interval (red segment) are grouped together. In this example, six groups are sorted out. The number of neurons in each group is dependent on the spike count distribution and spike interval. The algorithmic implementation of this threshold adaptation scheme is described in Algorithm 1.

**Algorithm 1:** Online adaptive pruning over time and neurons.

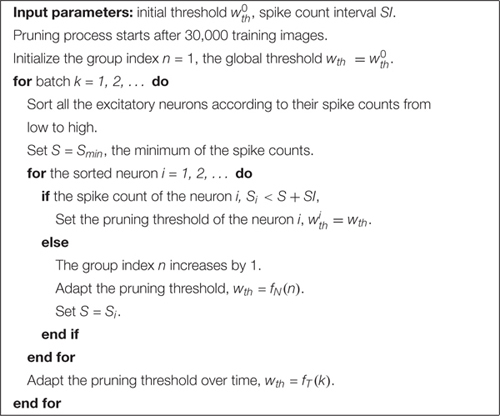

**FIGURE 6:**
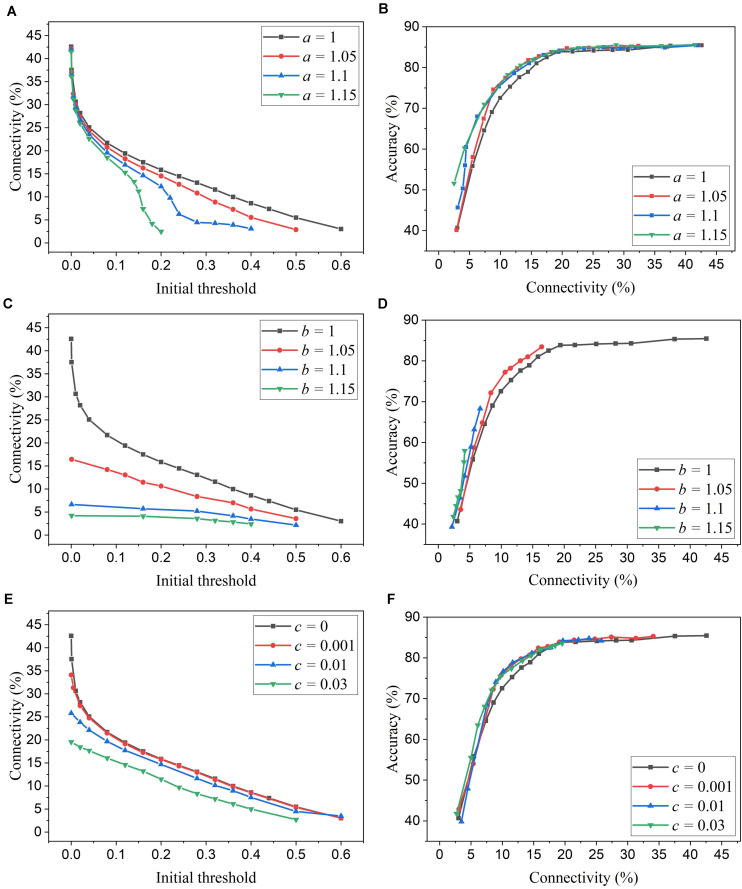
Simulation results of the online adaptive pruning over neurons for different adaptation functions in the SNN trained on MNIST dataset with 100 excitatory neurons. **(A,C,E)** show the network connectivity changes with the initial threshold for the adaptation functions *f*_*1*_, *f*_*2*_, and *f*_*3*_, respectively. **(B,D,F)** present the accuracy changes with the connectivity for the adaptation functions *f*_*1*_, *f*_*2*_, and *f*_*3*_, repectively. The connectivity is defined as the percentage of the unpruned weights. The spike interval is set as 30.

We simulated SNNs with the proposed online pruning method that uses the three different adaptation functions, namely *f*_*1*_, *f*_*2*_, and *f*_*3*_. Different values of the adaptation factor (*a*, *b*, and *c*) associated with each function were used in the simulation. The results are presented in Figure 6, including connectivity vs. initial threshold and accuracy vs. connectivity. In the simulation, the spike count interval is fixed as 30 to study the impact of different adaptation functions. In Figures 6A,C,E, a higher threshold leads to smaller connectivity, and a larger adaptation factor requires a smaller threshold to reach certain connectivity. Similar observations to those described in the case of adaptation over time can also be seen. In Figures 6B,D,F, for the three different adaptation functions, the optimized values of the corresponding adaptation factors can be selected as *a* = 1.15, *b* = 1.05, and *c* = 0.03, respectively.

### APTN: Online Adaptive Pruning Over Time and Neurons

In the adaptation scheme over time, the threshold changes with time, but all the neurons share the same threshold. In contrast, the adaptation scheme over neurons considers the spatial difference of firing activities of all the neurons and adapts the threshold over them, but the thresholds for neuron groups are constant over time. A full adaptation scheme combines these two schemes by adapting the pruning threshold for each neuron over time during training. The algorithmic implementation is described in Algorithm 1. The pruning process starts after training over 30,000 images, and a global pruning threshold is initialized as $w_{t\,h}^{0}$. At each pruning step, all the neurons are sorted according to their spike counts in the order from low counts to high counts. Neurons are grouped according to the grouping method described in section “APN: Online Adaptive Pruning Over Neurons.” The global threshold is first assigned to the first neuron group (G0). It is then adapted according to the adaptation function *f*_*N*_(*n*), and assigned to the following groups. After the adaptation process over neurons is completed, the global threshold is reset and updated with the time adaptation function *f*_*T*_(*k*), where *k* is the pruning-step index. This combined method takes into consideration both the time evolution of synaptic weights and the spatial difference of firing activity of neurons during training.

## Comparisons and Discussion

### Comparison Among the Proposed Weight Pruning Methods

**FIGURE 7:**
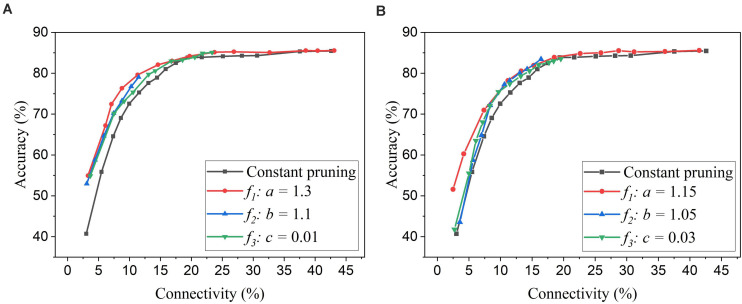
Performance comparison among different adaptation functions in the SNN trained on MNIST dataset with 100 neurons. **(A)** APT: Online adaptive pruning over time, and **(B)** APN: Online adaptive pruning over neurons. The spike count interval is set as 30.

**FIGURE 8:**
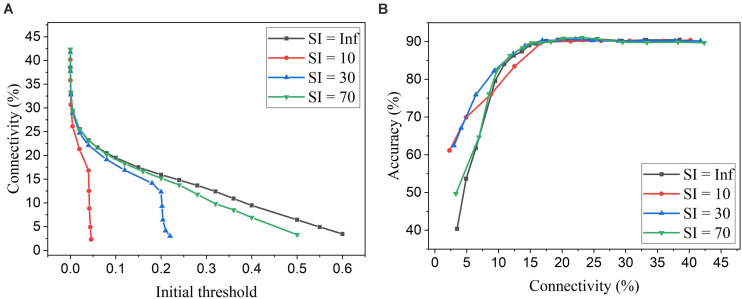
Simulation results of online adaptive pruning over neurons for different spike count intervals (SI) in the SNN trained on MNIST dataset with 100 neurons. **(A)** Connectivity vs. initial threshold, and **(B)** Accuracy vs. connectivity. SI = Infinity (Inf) means that there is only one group and hence no adaptation over neurons.

Firstly, we will compare the three different adaptation functions and select the best spike count interval. Figure 7A shows the comparison among the three adaptation functions with the optimized adaptation factors for the APT method. Clearly, the function *f*_*1*_ gives the best performance improvement over the constant pruning method, i.e., the highest accuracy when connectivity is smaller than 15% and similar accuracy to other functions otherwise. This can be attributed to the fact that *f*_*1*_ allows the pruning threshold to grow slowly at the early phase of the pruning process and hence more weights to be trained. It increases the threshold rapidly at the end, which guarantees largely reduced network connectivity. Figure 7B shows the comparison among the three adaptation functions with the optimized adaptation factors for the APN method. The same conclusion can be drawn that the function *f*_*1*_ gives the best performance. Moreover, after selecting the adaptation function as *f*_*1*_, we studied the effect of the spike count interval on the performance. The results are shown in Figure 8. The spike count interval is used to identify how similar the firing activities of neurons in the same group are. In Figure 8A, with a smaller interval, a smaller initial threshold is needed to reach a certain threshold. A small interval results in a large number of groups and hence creates a large difference in pruning threshold among the groups. This can cause a very high threshold to be applied in the group with weak neurons and deteriorate their performance significantly. So, it is not an effective grouping. A large interval can gather the neurons with very different firing activity into one group where the same pruning threshold is shared. This way is also not effective because a large threshold can significantly deteriorate the performance of weak neurons and a small threshold is not able to remove enough non-critical weights from strong neurons. From the results in Figure 8B, SI = 30 shows the best performance.

**FIGURE 9:**
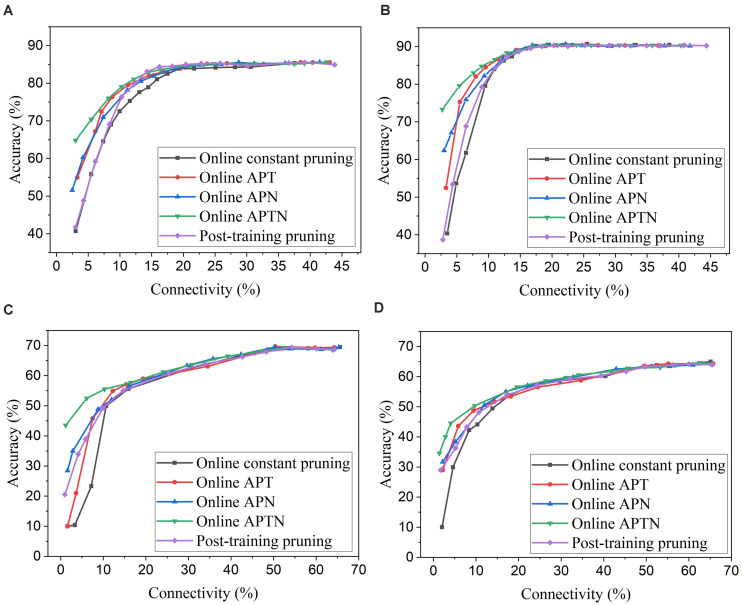
Comparison among different weight pruning methods in the SNN trained on different datasets. MNIST dataset: **(A)** 100 excitatory neurons and **(B)** 800 excitatory neurons. Fashion-MNIST dataset: **(C)** 100 excitatory neurons and **(D)** 800 excitatory neurons.

**FIGURE 10:**
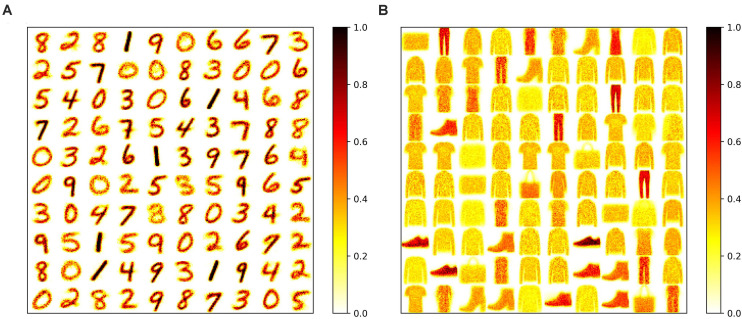
Trained weight maps on **(A)** MNIST dataset and **(B)** Fashion-MNIST dataset in the SNN with 100 neurons without pruning. Each pattern in the maps is formed by arranging the weights associated with each neuron to a 28 × 28 matrix.

To apply adaptation over both time and neurons, we combined the proposed adaptive pruning methods with the selected adaptation functions and adaptation factors. In this approach, the pruning threshold is increased over time and adapted across all the excitatory neurons. The comparisons among the proposed adaptive pruning methods for MNIST dataset and Fashion-MNIST dataset are shown in Figures 9A,C obtained from the SNN with 100 excitatory neurons, respectively. The same adaptation function and parameters were used to obtain the pruning results on Fashion-MNIST dataset. For all the pruning methods, up to 80% of weights trained on MNIST dataset can be pruned with less than 1% accuracy loss. It is because the trained weight maps on MNIST dataset are very sparse, as shown in Figure 10A. Whereas, only up to 50% of weights trained on Fashion-MNIST dataset can be pruned with less than 1% accuracy loss since the input patterns from Fashion-MNIST dataset are more complex, as shown in Figure 10B. Clearly, applying adaptation over both time and neurons can further improve the network performance, especially when the network becomes very sparse (connectivity < 10%). When the sparsity of the network increases, the performance of each excitatory neuron is very sensitive to critical weights, so it is very important for a pruning method to effectively identify critical weights and prevent the neurons from failure. The effect of the threshold adaptation lies in two different aspects. The first one is to allow the network to reserve critical weights when the network is not trained enough in the early phase of training. The second aspect is to balance the connection strength of excitatory neurons in the network so that more weights can be pruned from strong neurons and less from weak neurons to avoid causing substantial performance degradation of some neurons since neurons are critical processing units in the network. Moreover, a post-training pruning method is included for comparison (Rathi et al., 2019). Instead of pruning weight while training, this method prunes weights after the training process is done. It shows slightly better performance than the online constant pruning method but much worse performance than the proposed online APTN method when the connectivity is smaller than 10%. This is because, during the online constant pruning process, some critical weights can be mistakenly removed, whereas the adaptive method can effectively reserve the critical weights and provide more chances for them to be trained. The proposed pruning methods were also studied in the SNN with 800 excitatory neurons trained on both datasets. The adaptation function *f*_*1*_ was used. The time adaptation factor, the neurons adaptation factor, and the spike count interval were optimized and selected as 1.2, 1.15, and 30, respectively. The comparison among the proposed pruning methods is shown in Figures 9B,D. The pruning results show similar comparisons, and the same analysis can be applied. The post-training pruning shows slightly better performance than the online constant pruning method but worse performance than the proposed online adaptive pruning methods. The pruning results further confirm that the proposed APTN method outperforms the other weight pruning methods, especially when the network becomes very sparse (connectivity < 10%). We can draw a conclusion that the proposed APTN method is the most effective pruning method that can significantly reduce network connectivity and maintain high accuracy.

**FIGURE 11:**
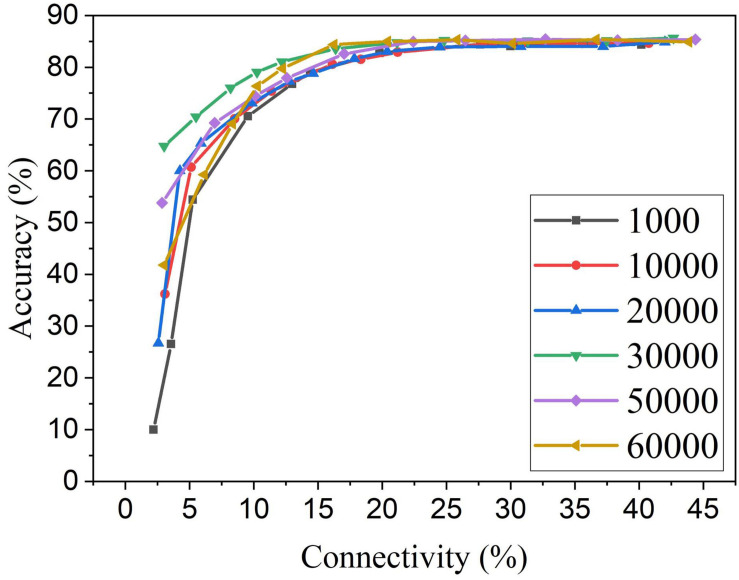
MNIST accuracy results at different connectivity values in the SNN with 100 neurons after applying APTN method. The number of pre-pruning training images was changed from 1,000, 10,000, 20,000, 30,000, 50,000, to 60,000.

For the proposed online pruning method, it is crucial to find the right starting point for pruning during training. If pruning starts too early, weights are not learned enough, and hence some critical weights can be mistakenly removed. While the weights are learned for enough time after 30,000 training images, the remaining training process will further fine-tune the unpruned critical weights as they get more chance for STDP updates when more weights are pruned. This is due to the dynamics of the STDP learning rule that the weights with more contribution to the neural firing are strengthened more often. So if pruning starts during the last stage of training, the unpruned weights will not have enough chance to be fine-tuned to preserve good network performance. The adaptation will not be carried out effectively, and there will be less improvement in accuracy and training energy efficiency. In section “Methods and results,” we decided to start pruning after training over 30,000 images based on the change of network dynamics. To further investigate the impact of the number of pre-pruning training images, we have obtained pruning results for the various number of pre-pruning training images which are presented in Figure 11. It confirms that pruning too early causes more performance loss. If the pruning happens at the later stage of training (50,000 pre-pruning training images), the performance loss is also observed, as there is almost no adaptation effect. 30,000 is proven to be the optimal point where neurons start to fire stably, and weight updates start to stabilize. Different from the post-training pruning method (60,000 pre-pruning training images), the online APTN method requires a crucial starting point during training in order to achieve the best network performance, and it also provides more chance for the unpruned critical weights to be trained during the training process. Starting at the 30,000 point, the online APTN method outperforms the post-training pruning method, especially when the network becomes very sparse, as demonstrated before.

Additionally, the selection of an adaptation function and the corresponding adaptation factors can be further optimized with more choices of functions and a finer grid of factor values. However, this is not in the scope of this work that aims to demonstrate the effectiveness of the proposed adaptive pruning method.

### Computational Cost Reduction

**FIGURE 12:**
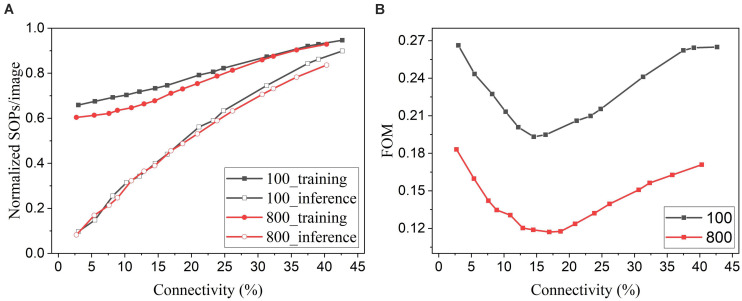
**(A)** Normalized SOPs/image and **(B)** a figure of merit (FOM) for different connectivity values are obtained in the SNNs with 100 and 800 excitatory neurons using the online adaptive pruning over time and neuron method.

In general, for neuromorphic hardware systems, like TrueNorth, SpiNNaker, and Loihi, the fundamental operation is the synaptic event that occurs when a spike is transmitted from a source neuron to a target neuron. So the computational energy of an SNN is proportional to the synaptic activity (Merolla et al., 2014). Pruning leads to a reduced number of synapses in the network and hence less synaptic events. To evaluate the energy improvement benefit of our proposed adaptive pruning method, we computed the number of SOPs per image (SOPs/image) during both training and inference. The training SOPs include weight accumulations and STDP updates, while the inference SOPs only count weight accumulations. The results for the SNNs trained on MNIST dataset with 100 and 800 excitatory neurons are shown in Figure 12A. The SOPs/image is normalized to the value obtained from the SNN without pruning. Clearly, the SOPs/image during both training and inference decreases almost linearly with connectivity, as the number of synaptic events is proportional to the number of unpruned synapses. The inference SOPs/image is reduced more significantly than the training SOPs/image. Moreover, the online pruning method can effectively reduce the number of training SOPs and hence improve training energy efficiency, making it promising for improving online learning systems. To help choose the network connectivity to reach the best overall performance, we define a figure of merit by considering accuracy loss and the total SOPs/image (training + inference) as below.

F⁢O⁢M=A⁢c⁢c⁢u⁢r⁢a⁢c⁢y⁢l⁢o⁢s⁢s×N⁢o⁢m⁢a⁢l⁢i⁢z⁢e⁢d⁢t⁢o⁢t⁢a⁢l⁢S⁢O⁢P⁢s/i⁢m⁢a⁢g⁢e

The defined FOM is used on a per-network basis to help identify the best network connectivity for that specific network, as demonstrated in Figure 12B. As a result, the best choices of the connectivity are 14.5% and 17% for 100-neuron and 800-neuron networks, respectively. Specifically, at 14.5% connectivity, the adaptive pruning method leads to a 27% reduction in SOPs/image during training and a 60% reduction during inference with 2.85% accuracy loss in the SNN with 100 excitatory neurons. In the case of 800 excitatory neurons, at 17% connectivity, the method leads to a 30% reduction during training and a 55% reduction during inference with only 0.44% accuracy loss. It should be noted that the proposed FOM provides one way to determine the best network connectivity, and other factors or definitions could also be applied depending on the requirements of specific applications.

### Comparison With Prior Works

**FIGURE 13:**
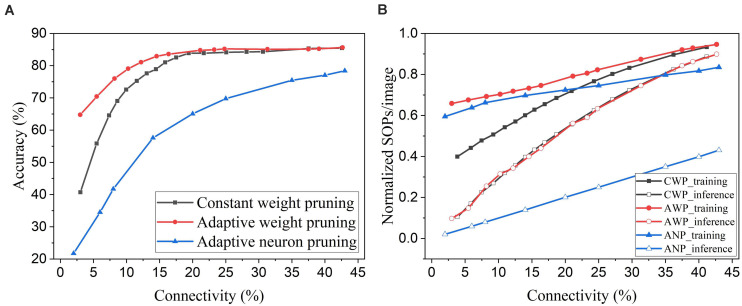
Comparison between online weight pruning methods and an online adaptive neuron pruning method in the SNN trained on MNIST dataset with 100 excitatory neurons. **(A)** Accuracy and **(B)** normalized SOPs/image change with connectivity. CWP, AWP, and ANP are short for constant weight pruning, adaptive weight pruning, and adaptive neuron pruning, respectively.

Neuron pruning is one of the structured weight pruning strategies, which eliminates all the weights associated with the pruned neurons and reduces the network complexity proportionally. However, directly removing neurons from the network could cause severe deterioration of network performance. We compared the proposed online weight pruning methods with an online adaptive neuron pruning method presented in our previous work (Guo et al., 2020). The comparison is shown in Figure 13. In Figure 13A, the online adaptive neuron pruning method shows worse accuracy than the weight pruning methods, which proves that weight pruning is more effective in preserving network performance. Despite the severe accuracy drop, the neuron pruning method requires fewer training SOPs/image than the adaptive weight pruning method and can reduce the inference SOPs/image much more significantly. Moreover, an additional benefit of the neuron pruning method is the elimination of state memory and processing power of the pruned neurons.

**FIGURE 14:**
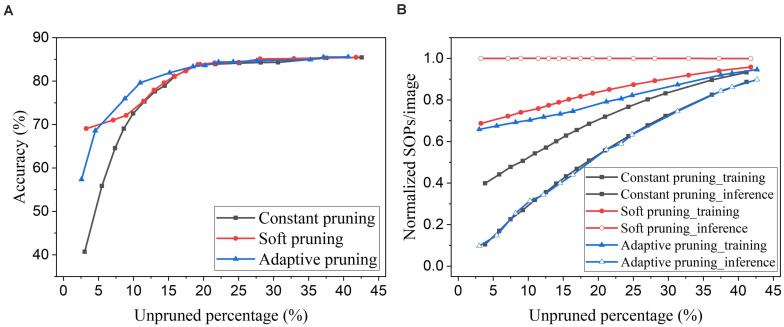
Comparison with the online soft weight pruning method adopted from Shi et al. (2019) in the SNN trained on MNIST dataset with 100 excitatory neurons. **(A)** Accuracy and **(B)** normalized SOPs/image change with unpruned weights percentage. Since the soft pruning method does not remove the pruned weights, the connectivity is not applicable as the *x* axis here. Instead, the unpruned percentage is used, which is defined as the percentage of the unpruned weights in the total weights before pruning.

An online soft weight pruning method for unsupervised SNNs was reported in Shi et al. (2019). Unlike conventional pruning methods, instead of removing the pruned weights, this method sets the pruned weights constant at the lowest possible weight value or the current value and stops updating them for the rest of the training process. By setting the pruned weights to the lowest possible value, the soft pruning method is equivalent to the constant pruning method in our case since the lowest value is 0. In this comparison, we refer to the soft pruning method as the case where the pruned weights are kept constant at their current values. Since the soft pruning method does not induce the sparsity in the network, the connectivity remains 100% and hence is not applicable in the comparison. Instead, we use the unpruned percentage that is the percentage of the unpruned weights in the total weights before pruning. In Figure 14A, it can be seen that the soft pruning method starts to have performance improvement over the constant pruning method after the unpruned percentage drops below 10%. Our proposed adaptive pruning method gives better performance when the unpruned percentage is between 5% and 20%, but worse performance after the unpruned percentage drops below 5%. When most of the weights are pruned, the soft pruning method is still able to retain high accuracy by keeping the pruned weights that were trained for some time in the network. However, the soft pruning method brings less benefit to the computational cost compared with the adaptive pruning method. Figure 14B shows that it contributes to less reduction in training SOPs/image and no reduction in inference SOPs/image. In comparison, our proposed adaptive pruning method can lead to more reduction in SOPs/image, especially during inference. The constant pruning method gives the most improvement in decreasing the training SOPs/image when a large number of weights are pruned at the cost of severe accuracy loss, because it applies a large constant threshold throughout the whole pruning process.

Comprehensive comparisons among different pruning methods in terms of accuracy loss and SOPs are provided in Algorithm 1, Table 3, including results for two network sizes and two datasets. The reduction is defined as the reduced percentage of the SOPs/image by pruning against the SOPs/image in the SNN without pruning. Network connectivity is selected as 10%. The neuron pruning method achieves the highest reduction in inference SOPs but the worst accuracy loss on both datasets. The post-training weight pruning method is able to produce small accuracy loss but no reduction in training SOPs. The soft online pruning method leads to the least accuracy loss on Fashion-MNIST dataset, because classifying more complex patterns in the dataset is more sensitive to the weights loss and this pruning method keeps the pruned weights in the network at their current values instead of removing them. However, this method leads to no benefits in reducing inference operations. The constant online pruning method can reduce both training and inference operations effectively at the cost of high accuracy loss. Our method achieves the least accuracy loss on MNIST dataset and slightly higher accuracy loss on Fashion-MNIST dataset than the soft online pruning method. Our method can lead to a large reduction in SOPs comparable to the constant online weight pruning and adaptive online neuron pruning methods during both training and inference. The network size has no substantial impact on the comparisons. In conclusion, our proposed adaptive pruning method can significantly reduce computational operations during both training and inference and maintain high accuracy at the same time.

**TABLE 3:** Comparison among different pruning methods in the SNN trained on Fashion-MNIST dataset.

Pruning methods	Accuracy loss 100/800	Training SOPs reduction 100/800	Inference SOPs reduction 100/800
Online adaptive neuron pruning Guo et al., 2020	42.14%/40.88%	23%/39%	90%/90%
Post-training weight pruning Rathi et al., 2019	16.35%/19.98%	0%/0%	82%/85%
Online soft weight pruning Shi et al., 2019	12.23%/9.63%	27%/29%	0%/0%
Online constant weight pruning Shi et al., 2019	20.43%/19.24%	45%/49%	88%/87%
Online adaptive weight pruning (Our work)	14.09%/13.67%	28%/31%	85%/84%

*Accuracy loss and SOPs reduction for two network sizes (100 and 800 neurons) are shown. The connectivity is selected as 10%.*

### Implementation Overhead

The proposed adaptive pruning algorithm can be implemented in hardware systems without adding significant overhead. To investigate the overhead, we chose three metrics: processing speed, area, and energy.

**FIGURE 15:**
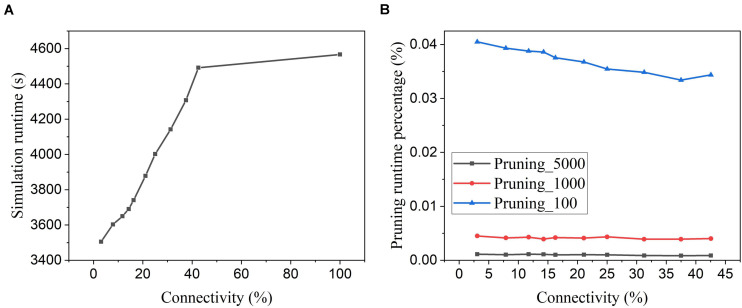
Simulation runtime. **(A)** Total network simulation runtime during training at different network connectivity values after applying the proposed adaptive pruning method APTN. **(B)** Pruning algorithm runtime percentage over the total network simulation time at different network connectivity values. Different batch sizes were used as 100, 1,000, and 5,000.

Figure 15A shows the software simulation runtime of the whole network during training, including the time used for executing the pruning algorithm. The software simulation is programmed in Python language and runs sequentially in a single process. The runtime decreases with the increasing pruning percentage (decreasing connectivity), which proves that the proposed online pruning method is able to shorten the network runtime as it reduces the number of SOPs, including weight accumulations and STDP updates. Besides, the APTN pruning runtime is negligible compared to the total runtime (SNN runtime plus pruning runtime). For example, in Figure 15B, the pruning runtime percentage is around 0.001% at the batch size of 5,000 and less than 0.04% even when the batch size is decreased to 100. For hardware runtime, we estimated the number of clock cycles required to run the pruning algorithm in a general synchronous digital system, as shown in Table 4. At each batch, the proposed pruning process requires three essential phases, including dividing the neuron groups (grouping phase), adapting pruning thresholds over neurons (adapting phase), and writing 0 s to weight memory (weight pruning phase) operations. The grouping method with sorting in the proposed algorithm can be replaced by simply searching for the minimum and maximum values of firing activities of neurons and dividing the whole range of firing activity (max – min) according to the spike interval without performance loss. The adapting phase is simply to position each neuron in the right group according to its firing activity and assign the corresponding pruning threshold. Both grouping and adapting phases depend on the number of groups that varies over time but is smaller than 20. We used 20 for the estimation. For both phases, we assume that no parallelism is applied for estimating the upper limit. Moreover, we assume that all the weights are stored in one memory, and the weight pruning operations can only access one weight at a time. However, it should be noted that multiple accesses to weight memory are available in practice. So the estimation is at the upper limit of the running cycles of the pruning algorithm. The estimated number of different phases in the table is for single-batch pruning. The number of clock cycles for the SNN training phase is much larger than the number of training SOPs/image, 96,099, since each SOP includes many processes, such as searching for destination addresses, reading out synaptic weights, routing spikes to the destination, and weight addition or STDP update, which takes multiple cycles to finish. It can be seen that the average number of clock cycles per image for pruning with a small batch size of 100 is far smaller than the number of training SOPs. Therefore, the hardware runtime of the pruning algorithm is negligible.

**TABLE 4:** Estimated number of clock cycles and computational operations (Ops) for the pruning algorithm and SNN training phase in the network with 100 neurons.

Phase	Pruning (single batch)			Pruning (average per image) Batch: 100/5,000	SNN Training (average per image)
	Grouping	Adapting	Weight pruning		
# Cycles	120	2,000	78,400	404/9	≫96,099
# Ops	400	1,100	156,800	794/18	96,099

*Two batch sizes (100 and 5,000) were used for estimating the average per image. The number of operations for SNN training only includes synaptic operations obtained at the connectivity of 10%.*

For energy overhead, the number of basic operations, such as addition, comparison, and memory access, was estimated in Table 4 for different pruning phases. For the estimation, 16 bits and 8 bits were used to represent the integer part and fractional part, respectively. The multiplication operation involved in the algorithm can be approximated by shift and addition operations. The grouping phase requires addition, comparison, and memory access operations, while the adapting phase only needs comparison and memory access operations. The operations in the weight pruning phase involve memory access and comparison between weights and a pruning threshold. The average number of operations per image is 794 and 18 for the batch size of 100 and 5,000, respectively, which are very small compared to the number of training SOPs/image. For energy comparison, we take an example of SNN implementation on Loihi neuromorphic hardware (Davies et al., 2018). The reported minimum energy/SOP on this hardware is 23.6 pJ. So the minimum SOP energy per image is around 2.3 uJ. Since memory access consumes more energy than addition and comparison operations, we used the energy of memory access for all the operations for the comparison. The memory access (read and write) to an SRAM cell under the same technology consumes around 0.5 pJ (Yang et al., 2016). So, the estimated energy for pruning operations per image is 3.4 nJ at the batch size of 100, which is around 0.1% of the SOP energy. Besides, the network also spends energy on updating neural states in neural cores, which makes the percentage even smaller. Thus, we can claim that the energy overhead is negligible.

As for area overhead, the number of essential digital gates and memories required to implement the pruning algorithm and equivalent NAND gates was estimated in Table 5. Each weight needs a flag bit to indicate if it has been pruned. This bit can be simply attached to the weight bits in the memory with very little overhead. The number of NAND gates for an SNN with 100 neurons was estimated according to the proposed digital implementation from Guo et al. (2020). Clearly, the number of equivalent NAND gates for the pruning algorithm is much smaller than that for the SNN. For example, the number of equivalent NAND gates in the pruning unit is only around 0.3% of that in the SNN. For memory comparison, in the pruning unit, firing activity and pruning threshold of neurons are assumed to be stored in block RAMs (BRAMs). Two 18 Kb BRAMs are totally enough, which is much smaller than the memory size required in the SNN. Therefore, the area overhead is very small.

**TABLE 5:** Estimated number of essential digital gates and memories required for the pruning algorithm and equivalent NAND gates.

Pruning unit					SNN	
Sub/Add (16 bits)	Comparator (16 bits)	Register	NAND	BRAM (18 Kb)	NAND	BRAM (18 Kb)
4	18	620	8284	2	3.0 × 10^6^	50

*The number of NAND gates and BRAMs for an SNN were obtained with 100 neurons according to the proposed digital implementation from (Guo et al., 2020).*

### Impact and Future Work

The proposed adaptive method would be effective in improving the compression rate and preserving good network performance in other neural networks, as different threshold adaptation techniques have also been applied to improve the pruning performance in other neural networks.

The iterative pruning method has been the most successful and popular pruning technique in ANNs, which relies on numerous cycles of training and pruning in order to induce sparsity in weight matrices and preserve network performance (Han et al., 2015). This method iteratively sets the weights below a certain threshold to zero and retrains the network to regain its performance. The main limitation is the need to manually tweak the thresholds for neurons in different layers to achieve the best results by iterative tuning. While this iterative method can effectively compress networks, it requires a large amount of time and resources in order to find the optimized sparse networks, which hinders its use in large-scale applications.

In order to eliminate the need for iterative threshold tuning, many works have explored to adapt threshold values for neurons in different layers by training the thresholds together with weights (Manessi et al., 2018; Ye et al., 2019; Azarian et al., 2020). These methods use the same concept of adapting threshold spatially as in our method based on the fact that neurons in different layers have different sensitivity to pruning thresholds, but in a different adaptation process. In our method, we used the firing activity of neurons to determine their pruning thresholds, while these methods adapt the thresholds based on the network loss in a supervised fashion. These methods were able to find the optimal thresholds for each layer and do not require pruning-retaining cycles. The results have shown that with the threshold adaptation, their methods can achieve a much larger compression rate with higher classification accuracy than the method without adaptation. Moreover, threshold adaptation over time during training was demonstrated to be beneficial in accelerating the pruning process and achieving a higher compression rate. Narang et al. (2017) proposed to adapt the pruning threshold over time using a monotonically increasing function during training. A heuristic function was presented to calculate the threshold at different iteration steps, which requires many hyper-parameters. They tested the method in different types of recurrent neural networks (RNNs) and demonstrated that this adaptive method could achieve better network performance and a higher compression rate without pruning-retraining cycles than a hard pruning method that simply prunes the weights with a constant threshold. Therefore, we believe that the proposed adaptive pruning method can be useful in improving the compression rate and preserving good network performance in ANNs. To test the versatility of our method, we will investigate the impact of the proposed adaptive method in deep SNNs in our future works.

## Conclusion

In this work, we proposed an online adaptive weight pruning method that adapts the pruning threshold over time and neurons during training in an unsupervised SNN. The effects of the threshold adaptation over time and neurons were studied individually. Different functions used to adapt the threshold were applied and compared. It is demonstrated that both adaptation over time and neurons can improve the network performance against an online constant weight pruning method. The adaptation enables the network to reserve critical weights when the network is not trained enough at the early phase of training and balance the connection strength of excitatory neurons in the network to avoid largely deteriorating the performance of weak neurons. So, combining the two adaptation schemes can further improve network performance. The online adaptive pruning method provides better performance than the post-training pruning method, suggesting that it can not only improve training energy efficiency but also achieve higher accuracy. Regarding the computational cost, the number of SOPs was analyzed, which shows that the proposed online adaptive pruning method can significantly reduce the SOPs/image during both training and inference. Furthermore, comparisons with the previous works reveal that our method can lead to better accuracy and a more significant reduction in SOPs. The implementation overhead of the proposed method was evaluated in terms of processing speed, area, and energy, which is proven to be negligible in the network. Therefore, the proposed online adaptive pruning method provides a promising approach for reducing network complexity and improving energy efficiency with good performance in SNNs for real-time applications.

## Data Availability Statement

The original contributions presented in the study are included in the article, further inquiries can be directed to the corresponding author.

## Author Contributions

WG, MF, HY, AE, and KS: conceptualization. WG, MF, HY, AE, and KS: methodology. WG: software, algorithms and writing – original draft preparation. WG, MF, and HY: investigation and validation. MF, HY, AE, and KS: writing – review and editing. AE and KS: supervision. KS: project administration. All authors contributed to the article and approved the submitted version.

## Conflict of Interest

The authors declare that the research was conducted in the absence of any commercial or financial relationships that could be construed as a potential conflict of interest.

## References

[B1] Anwar S. Hwang K. Sung W. (2017). Structured pruning of deep convolutional neural networks. *J. Emerg. Technol. Comput. Syst.* 13:32 10.1145/3005348

[B2] Azarian K. Bhalgat Y. Lee J. Blankevoort T. (2020). Learned threshold pruning. *ArXiv* [Preprint]. Available online at: https://arxiv.org/pdf/2003.00075.pdf (accessed October 4, 2020).

[B3] Burkitt A. N. (2006). A review of the integrate-and-fire neuron model: i. homogeneous synaptic input. *Biol. Cybernet.* 95 1–19. 10.1007/s00422-006-0068-6 16622699

[B4] Davies M. Srinivasa N. Lin T. Chinya G. Cao Y. Choday S. H. (2018). Loihi: a neuromorphic manycore processor with on-chip learning. *IEEE Micro* 38 82–99. 10.1109/MM.2018.112130359

[B5] Diehl P. Cook M. (2015). Unsupervised learning of digit recognition using spike-timing-dependent plasticity. *Front. Comput. Neurosci.* 9:99. 10.3389/fncom.2015.00099 26941637 PMC4522567

[B6] Fontaine B. Peña J. L. Brette R. (2014). Spike-threshold adaptation predicted by membrane potential dynamics in vivo. *PLoS Comput. Biol.* 10:e1003560. 10.1371/journal.pcbi.1003560 24722397 PMC3983065

[B7] Furber S. B. Galluppi F. Temple S. Plana L. A. (2014). The SpiNNaker project. *Proc. IEEE* 102 652–665. 10.1109/JPROC.2014.2304638

[B8] Guo W. Yantır H. E. Fouda M. E. Eltawil A. M. Salama K. N. (2020). Towards efficient neuromorphic hardware: unsupervised adaptive neuron pruning. *Electronics* 9:1059. 10.3389/fnins.2020.598876 PMC7689062 33281549

[B9] Han S. Pool J. Tran J. Dally W. J. (2015). “Learning both weights and connections for efficient neural networks,” in *Proceedings of the 28th International Conference on Neural Information Processing Systems – Volume 1*, (Montreal: MIT Press).

[B10] Iglesias J. Eriksson J. Grize F. Tomassini M. Villa A. E. P. (2005). Dynamics of pruning in simulated large-scale spiking neural networks. *Biosystems* 79 11–20. 10.1016/j.biosystems.2004.09.016 15649585

[B11] Lecun Y. Bottou L. Bengio Y. Haffner P. (1998). Gradient-based learning applied to document recognition. *Proc. IEEE* 86 2278–2324. 10.1109/5.726791

[B12] Li H. Liu N. Ma X. Lin S. Ye S. Zhang T. (2019). “ADMM-based weight pruning for real-time deep learning acceleration on mobile devices,” in *Proceedings of the 2019 on Great Lakes Symposium on VLSI*, (Tysons Corner, VA: Association for Computing Machinery).

[B13] Manessi F. Rozza A. Bianco S. Napoletano P. Schettini R. (2018). “Automated Pruning for Deep Neural Network Compression,” in *Proceedings of the 2018 24th International Conference on Pattern Recognition (ICPR)*, Beijing, 657–664.

[B14] Mead C. (1990). Neuromorphic electronic systems. *Proc. IEEE* 78 1629–1636. 10.1109/5.58356

[B15] Merolla P. A. Arthur J. V. Alvarez-Icaza R. Cassidy A. S. Sawada J. Akopyan F. (2014). A million spiking-neuron integrated circuit with a scalable communication network and interface. *Science* 345:668. 10.1126/science.1254642 25104385

[B16] Narang S. Diamos G. Sengupta S. Elsen E. (2017). Exploring sparsity in recurrent neural networks. *ICLR*

[B17] O’Connor P. Neil D. Liu S.-C. Delbruck T. Pfeiffer M. (2013). Real-time classification and sensor fusion with a spiking deep belief network. *Front. Neurosci.* 7:178. 10.3389/fnins.2013.00178 24115919 PMC3792559

[B18] Paupamah K. James S. Klein R. (2020). “Quantisation and pruning for neural network compression and regularisation,” in *Proceedings of the 2020 International SAUPEC/RobMech/PRASA Conference*, Cape Town, 1–6. 10.1109/SAUPEC/RobMech/PRASA48453.2020.9041096

[B19] Pfister J.-P. Gerstner W. (2006). Triplets of spikes in a model of spike timing-dependent plasticity. *J. Neurosci.* 26 9673–9682. 10.1523/jneurosci.1425-06.2006 16988038 PMC6674434

[B20] Rathi N. Panda P. Roy K. (2019). STDP-based pruning of connections and weight quantization in spiking neural networks for energy-efficient recognition. *IEEE Trans. Comput. Aided Des. Integr. Circ. Syst.* 38 668–677. 10.1109/TCAD.2018.2819366

[B21] Shi Y. Nguyen L. Oh S. Liu X. Kuzum D. (2019). A soft-pruning method applied during training of spiking neural networks for in-memory computing applications. *Front. Neurosci.* 13:405. 10.3389/fnins.2019.00405 31080402 PMC6497807

[B22] Shrestha A. Mahmood A. (2019). Review of deep learning algorithms and architectures. *IEEE Access* 7 53040–53065. 10.1109/ACCESS.2019.2912200

[B23] Sredojevic R. Cheng S. Supic L. Naous R. Stojanovic V. (2017). Structured deep neural network pruning via matrix pivoting. *ArXiv* [Preprint]. Available online at: https://arxiv.org/abs/1712.01084#:~:text=In%20this%20work%20we%20introduce,for%20obtaining%20resource%2Defficient%20DNNs (accessed July 15, 2020).

[B24] Thakur C. S. Molin J. L. Cauwenberghs G. Indiveri G. Kumar K. Qiao N. (2018). Large-scale neuromorphic spiking array processors: a quest to mimic the brain. *Front. Neurosci.* 12:891. 10.3389/fnins.2018.00891 30559644 PMC6287454

[B25] Tung F. Mori G. (2020). Deep neural network compression by in-parallel pruning-quantization. *IEEE Trans. Pattern Anal. Mach. Intellig.* 42 568–579. 10.1109/TPAMI.2018.2886192 30561340

[B26] Xiao H. Rasul K. Vollgraf R. (2017). Fashion-MNIST: a novel image dataset for benchmarking machine learning algorithms. *ArXiv* [Preprint]. Available online at: https://arxiv.org/abs/1708.07747 (accessed October 11, 2020).

[B27] Yang Y. Jeong H. Song S. C. Wang J. Yeap G. Jung S. (2016). Single Bit-Line 7T SRAM cell for near-threshold voltage operation with enhanced performance and energy in 14 nm FinFET technology. *IEEE Trans. Circ. Syst. I Regul. Pap.* 63 1023–1032. 10.1109/TCSI.2016.2556118

[B28] Ye S. Feng X. Zhang T. Ma X. Lin S. Li Z. (2019). Progressive DNN compression: a key to achieve ultra-high weight pruning and quantization rates using ADMM. *ArXiv* [Preprint]. Available online at: https://arxiv.org/abs/1903.09769 (accessed October 4, 2020).

[B29] Zillmer E. A. Spiers M. V. (2001). *Principles of Neuropsychology.* Belmont, CA: Wadsworth/Thomson Learning.

